# Gad c 1 efficiency in the diagnosis of fish allergy in children

**DOI:** 10.1186/2045-7022-3-S3-P54

**Published:** 2013-07-25

**Authors:** O Domínguez, P Carrillo, M Giner, M Piquer, M Alvaro, R Jimenez-Feijoo, J Lozano, M Pascal, A Plaza

**Affiliations:** 1 Allergy and Clinical Immunology Department, Hospital Sant Joan de Deu, Barcelona, Spain; 2 Allergy Department, Hospital Virgen Arrixaca, Murcia, Spain; 3 Immunology Department, CDB. Hospital Clinic, Barcelona, Spain

## Background

Fish is one of the main culprit foods in childhood allergy [prevalence below 5 years 30% and then 9.8%, Spain]. IgE-mediated fish allergy diagnosis (FAD) is based on specific IgE (sIgE) detection to fish whole extracts and parvalbumin, considered to be major allergen, despite oral challenge (OC) is still the gold standard. Parvalbumin is an important panallergen. Cod parvalbumin (Gad c 1) is used for FAD in general; however its efficiency in FAD to species other than cod has not been reported. We sought to evaluate Gad c1 efficiency in FAD.

## Methods

Medical records of children screened and followed for suspected fish allergy from 2009 to 2012 were reviewed looking for clinical data, sensitization to frequent fish species (cod, hake, sole; monkfish, tuna, swordfish, sardine) and Gad c 1, and OC outcome. Descriptive statistics and Gad c 1 efficiency was reported. Patients were grouped: A (Gad c 1 +) and B (-).

## Results

Data of 58 children (median[range] age: 7 [4-17]; 57.6% boys) was collected. 75.8% had multiple food allergy, 69.5% respiratory and 38% atopic dermatitis (AD). 9 (15%) had Gad c 1 sensitization. Regarding symptoms at diagnosis: anaphylaxis (An, 28%) [hake (34%)/sole (15%)], urticaria (24%) and AD (12%). 41% have had symptoms in several episodes and different species. 5 (10%) had symptoms by inhalation. 40 (69%) belonged to A and 18 to B. OC data about 22 of A showed: for blue fish, tuna was usually the first one challenged and the most frequently tolerated (T) [canned (C, n=19: 18 T/1 An) and fresh (F, n=11, 9 T)]. For swordfish (n=8: 7 T/1 An) and sardine was tried in 1 causing An. For white fish, hake was the most frequent OC (n=4: 1 T/3 An). For sole (n=3: 1 T), monkfish (n=3, 3 An) and cod was tried in 1 patient causing An. In B, OC data was available on 7 children (39%) and tolerance rate was globally higher than A. For blue fish, tuna (F/C, n=3) all tolerated, swordfish (n=0) and sardine (n=1, T). For white fish, hake was the most frequently used for OC (n=6, all T). For sole (n=5, 4 T) and monkfish (n=5, all T). Cod was tried in none. Gad c 1 efficiency was for: hake (90%), sole (75%), tuna (36% F/18% C). Efficiency for swordfish, sardine, monkfish and cod could not be evaluated because of too small sample size.

## Conclusion

Hake and sole were the most frequent offending species. Most children present anaphylaxis and urticaria (at diagnosis and OC outcome). Despite Gad c 1 is used for routine FAD, it seems to be more efficient in white fish allergy than blue fish.

## Disclosure of interest

None declared.

